# Impact of Intrauterine Growth Restriction on Cognitive and Motor Development at 2 Years of Age

**DOI:** 10.3389/fphys.2018.01278

**Published:** 2018-09-19

**Authors:** Julia Hartkopf, Franziska Schleger, Jana Keune, Cornelia Wiechers, Jan Pauluschke-Froehlich, Magdalene Weiss, Annette Conzelmann, Sara Brucker, Hubert Preissl, Isabelle Kiefer-Schmidt

**Affiliations:** ^1^Institute for Diabetes Research and Metabolic Diseases of the Helmholtz Center Munich at the University of Tuebingen, Tuebingen, Germany; ^2^German Center for Diabetes Research (DZD e.V.), Tuebingen, Germany; ^3^fMEG Center, University of Tuebingen, Tuebingen, Germany; ^4^Department of Neurology, Klinikum Bayreuth GmbH, Bayreuth, Germany; ^5^Department of Neonatology, University of Tuebingen, Tuebingen, Germany; ^6^Department of Women’s Health, University of Tuebingen, Tuebingen, Germany; ^7^Department of Child and Adolescent Psychiatry, Psychosomatics and Psychotherapy, University of Tuebingen, Tuebingen, Germany; ^8^Department of Internal Medicine, Division of Endocrinology, Diabetology, Angiology, Nephrology and Clinical Chemistry, University of Tuebingen, Tuebingen, Germany

**Keywords:** intrauterine growth restriction, child development, fetal magnetoencephalography, visual event-related responses (VER), auditory event-related responses (AER)

## Abstract

Intrauterine growth restriction (IUGR), which is already known to be a risk factor for pathological intrauterine development, perinatal mortality, and morbidity, is now also assumed to cause both physical and cognitive alterations in later child development. In the current study, effects of IUGR on infantile brain function were investigated during the fetal period and in a follow-up developmental assessment during early childhood. During the fetal period, visual and auditory event-related responses (VER and AER) were recorded using fetal magnetoencephalography (fMEG). VER latencies were analyzed in 73 fetuses (14 IUGR fetuses) while AER latencies were analyzed in 66 fetuses (11 IUGR fetuses). Bayley Scales of Infant Development, Second Edition (BSID-II) were used to assess the developmental status of the infants at the age of 24 months. The Mental Development Index (MDI) was available from 66 children (8 IUGR fetuses) and the Psychomotor Development Index (PDI) from 63 children (7 IUGR fetuses). Latencies to visual stimulation were more delayed in IUGR than in small for gestational age (SGA) or appropriate for gestational age (AGA) fetuses, albeit not to any significant extent (*p* = 0.282). The MDI in former IUGR infants was significantly lower (*p* = 0.044) than in former SGA and AGA infants. However, IUGR had no impact on PDI (*p* = 0.213). These findings support the hypothesis that IUGR may constitute a risk factor for neurodevelopmental delay. Further investigation of the possible underlying mechanisms, as well as continued long-term developmental research, is therefore necessary.

## Introduction

Over the last few decades, it has become evident that events during early development in humans – even during the prenatal phase – can have long-term effects on health and disease. This concept is commonly known as Developmental Origins of Health and Disease (Barker, 2007; Wadhwa et al., 2009).

One trademark of anomalous prenatal development is intrauterine growth restriction (IUGR). Intrauterine growth restriction is characterized by a pathological restriction of fetal weight, as is presumed to be the case when a fetus is “small for gestational age” (SGA), i.e., when its estimated fetal weight and birth weight are below the 10th percentile for gestational age (GA). The literature and practice often does not distinguish clearly between IUGR and SGA. Consistent criteria are therefore required to establish general valid guidelines in diagnosis and treatment (Barker et al., 2013; Unterscheider et al., 2014; Levine et al., 2015). SGA, which is a more general term for those fetuses and infants whose estimated and actual birth weights are below the 10th percentile, is not necessarily connected with a pathological finding. The term also includes cases of below-average weight caused by genetic preconditions. By contrast, IUGR is associated with pathological intrauterine changes that cause restricted fetal growth. It is also linked to a higher risk of perinatal mortality and morbidity and requires appropriate medical support (Craigo, 1994; Bamberg and Kalache, 2004). It is important to distinguish between pathologically growth-restricted fetuses and constitutionally small fetuses. Placental insufficiency, the most frequently observed pathological cause for restricted fetal growth, should be diagnosed by the umbilical artery Doppler velocity (Figueras and Gardosi, 2011). Placental insufficiency is associated with metabolic and hormonal influences on the fetuses and manifests itself by reduced fetal growth and weight gain during pregnancy. These processes can lead to specific alterations in later physical and cognitive development known as “fetal programming” (Godfrey and Barker, 2001; Martin-Gronert and Ozanne, 2012). Since this influence begins during pregnancy, an early investigational approach is advisable.

Recent follow-up studies with former IUGR infants often used only reduced body size or abnormal Doppler for diagnosis of IUGR. A review by Murray et al. (2015) showed that only a small number of studies on the neurodevelopmental outcome in children with IUGR born at 35 weeks of gestation or later used both abnormal Doppler and small size as diagnostic criteria. The authors reported that IUGR is associated with an increased risk for neurodevelopmental delay. Children with fetal circulatory redistribution (i.e., a pathological Doppler) were reported to be more severely affected.

Neurodevelopmental impairments in IUGR infants are reflected by morphological and structural brain alterations and impaired brain function even in utero (D’Hooghe and Odendaal, 1991; Vindla et al., 1997; Nijhuis et al., 2000; Tolsa et al., 2004; Dubois et al., 2008; Lodygensky et al., 2008). In earlier trials, changes in body movements and heart rate were the two main indicators for stimulus processing for investigating the influence of IUGR on functional brain development in utero. Following acoustic or vibroacoustic stimulation, heart rate responses in IUGR fetuses were delayed and their body movement patterns lower than in controls (Gagnon et al., 1988, 1989; Kisilevsky et al., 2014).

Fetal magnetoencephalography (fMEG) is a non-invasive method for measuring fetal brain activity. From the GA of 28 weeks onward, fetal auditory event-related brain responses (AER) and visual event-related brain responses (VER) can be recorded and a decrease of latency can be assumed to be a marker of the maturation and integrity of functional fetal brain development (Schleussner et al., 2001; Eswaran et al., 2002; Schleussner and Schneider, 2004; Holst et al., 2005; Kiefer et al., 2008). Against this background, by demonstrating that IUGR fetuses have slower VER than their appropriate for gestational age (AGA) control counterparts, we recently ascertained that VER latency is associated with fetal outcome (Morin et al., 2015). A follow-up study to determine the impact of VER latencies on early childhood development, i.e., from birth to 24 months of age, is currently under way.

In the present study, we aimed to determine whether fetal outcome affects early childhood development. This entailed a developmental assessment using BSID-II that was performed at the age of 24 months in former IUGR, SGA, and AGA children. Furthermore, we investigated whether VER and AER latencies, as assessed by fMEG, differed between the fetal outcome groups.

## Materials and Methods

### Participants

One hundred and seven women with singleton pregnancies were recruited by the Department of Obstetrics and Gynecology at the University Hospital, Tuebingen. They gave written informed consent of their and their infant’s participation prior to the study, which was approved by the local Ethical Committee of the Medical Faculty of the University of Tuebingen (No. 476/2008MPG1). The study was performed in accordance with the relevant guidelines and regulations.

Fifteen of the infants had birth weights below the 10th percentile, and an increased umbilical artery pulsatility index above the 90th percentile for the respective GA was observed during pregnancy. These 15 fetuses were classified as IUGR due to an insufficient placental blood supply. Although 32 of the infants were born with weights below the 10th percentile, they had a normal umbilical artery Doppler during pregnancy and no placental insufficiency was found. These 32 fetuses were classified as constitutionally SGA. Sixty healthy children with an AGA birth weight were included as controls.

### fMEG Measurement

To investigate potential differences in brain development already during pregnancy, all participants underwent an fMEG measurement with visual and auditory stimulation to record event-related brain responses of the fetuses from 28 weeks of GA. The fMEG measurement was performed with a magnetoencephalographic system for fetal and neonatal studies (SARA II: SQUID Array for Reproductive Assessment, VSM MedTech Ltd., Port Coquitlam, BC, Canada). During the measurement, the woman placed her abdomen in an ergonomically shaped array containing 156 primary and 29 reference sensors. Visual stimuli were presented during 10 min of the measurement and consisted of light flashes delivered by fiber optic wire to an LED-light pad that was placed on the maternal abdomen near the location of a fetal eye, as determined via ultrasound. The light flashes had a wavelength of 625 nm and an intensity of 8000 lux; stimulus duration was 500 ms and the ISI was set at random between 1.5 and 2.5 s (Morin et al., 2015).

Auditory stimulation consisted of an oddball-paradigm with pure tones and was presented for a further 10 min of the measurement. Stimulus duration was 500 ms and the ISI was randomly selected between 1900 and 2100 ms. Standard tones, presented with a frequency of 500 Hz, were interspersed with deviant tones presented at 750 Hz to avoid habituation to the standard tone. Stimuli were delivered into a balloon via an air-filled tube placed on the maternal abdomen. The sequence of visual and auditory stimulation was randomized over subjects. Fetal data were recorded with a sampling rate of 610.352 Hz (Muenssinger et al., 2013).

### fMEG Data Analysis

Recorded fetal auditory and visual datasets were filtered offline with a high-pass filter of 0.5 Hz and were transformed by a first-order gradiometer to eliminate any external interference. Maternal and fetal heart signals were attenuated by signal space projection (McCubbin et al., 2006). The data was cut into segments ranging from 200 ms before to 1000 ms after the stimulus. A 10 Hz low-pass filter was applied and the average of the segments was calculated. VER and AER were analyzed by visual examination and defined as a peak in a time window of 80–500 ms after the stimulus, with a minimal amplitude of 4 fT in at least four sensors around the fetal head coil. The latency between stimulus onset and peak was documented for further statistical analysis.

### Developmental Test

**FIGURE 1:**
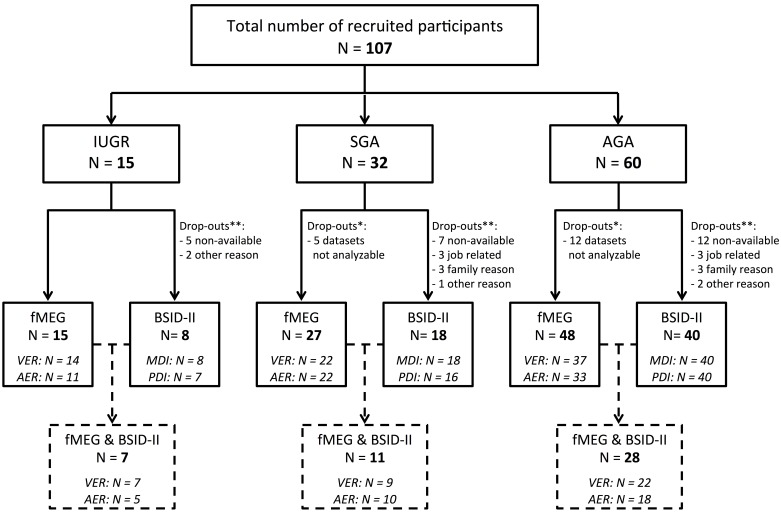
Flow chart showing enrollment and follow-up. All 107 infants were evaluated using fMEG. At the age of 2 years, developmental assessment was performed using BSID-II. ^∗^Indicates the number of fMEG data sets that were not analyzable. ^∗∗^Indicates the number of children that were lost to follow-up. At the bottom of the figure, the number of children for whom BSID-II and fMEG was available is shown.

Two years after fMEG measurement, all families were invited to participate in an assessment of their child’s development with BSID-II. Of a total of 107 participants, 66 returned for the follow-up assessment. The 41 participants who discontinued were distributed as follows: IUGR group: 7 of 15 children (46.7%), SGA group: 14 of 32 children (43.8%) and AGA group: 20 of 40 children (50%). The most common reasons for non-participation are summarized in **Figure 1**.

The BSID-II was developed for the measurement of the current developmental state of infants and children between 1 and 42 months of age (Nellis and Gridley, 1994). An experienced and trained psychologist, who was unaware of the medical history of the infant, conducted the test with the child in the presence of a parent. The BSID-II is divided into two scales: the MDI and PDI. The cognitive and psychomotor development of a child can therefore be assessed separately. MDI and PDI both have a mean of 100 and a standard deviation (SD) of 15.

### Statistics

Data was described as mean ± SD. A preliminary assumption check revealed that data was normally distributed, as assessed by Shapiro–Wilk test (*p* > 0.05), and that there were no univariate or multivariate outliers, as assessed by boxplot and Mahalanobis distance (*p* > 0.001), respectively. MDI, PDI, VER latency, and AER latency were analyzed for differences between fetal outcome groups (IUGR, SGA, and AGA) using one-way ANOVA and Welch’s test of unequal variances, respectively. *Post hoc* analyses were performed using the Games-Howell correction method. PASW Statistics 21 (SPSS Inc., Chicago, IL, United States) was used for statistical analysis and the significance level was set to *p* < 0.05.

## Results

Auditory event-related responses and VER latencies were measured using fMEG in 107 fetuses. Of these, 15 were IUGR fetuses, 32 were SGA fetuses and 60 were AGA fetuses. **Table 1** shows mean and SD for GA at birth and birth weight. VER latencies could be analyzed in a total of 73 fetuses (14 IUGR, 22 SGA, and 37 AGA) at a mean GA of 34.1 weeks. AER latencies were detectable in a total of 66 fetuses (11 IUGR, 22 SGA, and 33 AGA) at a mean GA of 34.0 weeks.

**Table 1:** Mean weeks (wks) of gestational age (GA) at the time of VER measurement, AER measurement and birth as well as birth weight in grams (g) of IUGR versus SGA versus AGA fetus.

Fetal group		GA at VER (wks)	GA at AER (wks)	GA at birth (wks)	Birth weight (g)
IUGR	Mean	33.6	33.8	35.5	1720
	N	14	11	15	15
	SD	2.7	2,6	2.8	405
SGA	Mean	34.7	34.1	38.6	2446
	N	22	23	32	32
	SD	3.0	3.4	2.1	401
AGA	Mean	33.9	33.9	40.0	3454
	N	37	34	60	60
	SD	3.0	3.1	1,6	412


**FIGURE 2:**
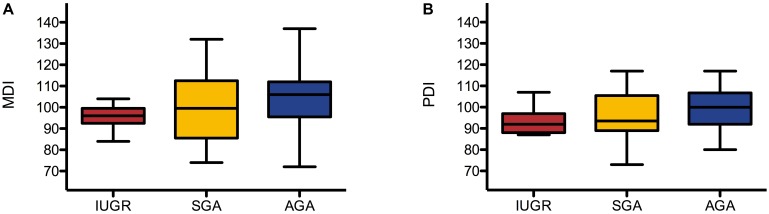
Box plots of MDI **(A)** and PDI **(B)** in IUGR, SGA, and AGA group. Box plots represent median, interquartiles, and ranges.

Mental Development Index and PDI were assessed using BSID-II at a mean (± SD) age of 24.10 (± 0.79) months. MDI was assessed in 66 children (8 IUGR, 18 SGA, and 40 AGA) and was 96 (± 6), 100 (± 16), and 103 (± 13), respectively. PDI was assessed in 63 children (7 IUGR, 16 SGA, and 40 AGA) and was 94 (± 7), 96 (± 11), and 100 (± 10), respectively. Results are presented as box plots in **Figure 2**.

**Table 2** shows the results of the univariate one-way ANOVA in a comparison of MDI, PDI, VER latency, and AER latency in the IUGR, SGA, and AGA groups. There were no statistically significant differences between PDI (*p* = 0.213), VER latency (*p* = 0.282), and AER latency (*p* = 0.206). However, the MDI differed significantly between groups (*p* = 0.044) and increased from the IUGR (96 ± 6) to the SGA (100 ± 16) as well as to the AGA group (103 ± 13). Games-Howell *post hoc* analysis (**Table 3**) revealed that the difference between IUGR and AGA was statistically significant (*p* = 0.035).

**Table 2:** MDI, PDI, VER latency and AER latency in IUGR, SGA, and AGA groups as calculated using one-way ANOVA.

		*N*	Mean	*SD*	*p*-value
MDI	IUGR	8	96	6	
	SGA	18	100	16	
	AGA	40	103	13	
	Total	66	101	13	0.044^∗^
PDI	IUGR	7	94	7	
	SGA	16	96	11	
	AGA	40	100	10	
	Total	63	98	10	0.213
VER latency	IUGR	14	233	59	
	SGA	22	217	64	
	AGA	37	204	57	
	Total	73	213	60	0.282
AER latency	IUGR	11	204	65	
	SGA	22	220	61	
	AGA	33	188	69	
	Total	66	201	67	0.206


*Welch’s t-test was used on account of unequal variances and sample-sizes and ^∗^indicates a significant group difference.*

**Table 3:** Comparisons of the MDI between the IUGR, SGA, and AGA groups.

	IUGR	SGA	AGA
IUGR	–	-4.44 (± 4.33) *p* = 0.568	-7.83 (± 2.91) *p* = 0.035
SGA	4.44 (± 4.33) *p* = 0.568	–	-3.38 (± 4.26) *p* = 0.710
AGA	7.83 (± 2.91) *p* = 0.035	3.38 (± 4.26) *p* = 0.710	–


*The differences of mean MDI values (± standard error) are shown and the respective p-values were calculated by Games-Howell *post hoc* analysis.*

## Discussion

In the current study, we aimed to investigate the impact of IUGR on early child development. At the age of two, children’s developmental status was assessed using BSID-II. The MDI was significantly lower in the IUGR than in the AGA group. Although scores for the PDI decreased from AGA to SGA, and IUGR, these differences were not statistically significant. In addition, fetal brain responses to visual and sound stimulation were assessed via fMEG before birth. We observed an increase in VER latencies from AGA over SGA to IUGR fetuses. These latency differences were, however, not statistically significant. Our results suggest that functional brain development maybe already altered during gestation and might cause an alteration in the neurological developmental trajectory in later life. However, it must be emphasized that these findings are based on a relatively small group of children.

Intrauterine growth restriction, a pathologic growth restriction of fetuses, is associated with significant neonatal morbidity and mortality (Nardozza et al., 2017). It is also believed to impact morphological and structural brain development (D’Hooghe and Odendaal, 1991; Vindla et al., 1997; Nijhuis et al., 2000; Tolsa et al., 2004; Dubois et al., 2008; Lodygensky et al., 2008). We recently reported that latencies of fetal AER and VER assessed by fMEG are delayed in fetuses with IUGR (Morin et al., 2015). In the present study, however, the differences in VER and AER latencies between IUGR, SGA, and AGA fetuses were not statistically different. A possible explanation for these seemingly contradictory findings may be due to the fact that we had used a case control approach in the previous study to match subjects for GA and fetal behavioral state. Since our primary focus in the present study was on the effect of IUGR on neurodevelopmental changes at 2 years of age, we decided to increase sample size by including not only matched pairs of SGA-AGA and IUGR-AGA subjects but also of all other subjects. For proof of the possible predictive value of fMEG, further studies with larger population sizes and longitudinal assessment of functional brain development are necessary.

In the current study, we used simple tone stimulation only. However, since several cognitive capabilities such as discrimination and habituation are already established in the last trimester of gestation, it would be worthwhile to apply these stimulation paradigms to determine whether they are more specific for alterations of early fetal brain development (Draganova et al., 2005; Matuz et al., 2012; Muenssinger et al., 2013; Hartkopf et al., 2016). Interestingly, intrauterine auditory stimulation with the maternal voice in growth-restricted fetuses has been proposed as a potential tool to compensate brain alterations that might be responsible for later language impairment (Kisilevsky et al., 2014).

When it came to childhood development, we observed lower cognitive and psychomotor abilities in IUGR than in AGA children, although only the differences in cognitive (mental) scores were of statistical significance. In line with our results, earlier trials showed that former IUGR infants are more liable to achieve lower scores in neurocognitive and/or motor developmental assessment tests than control children without IUGR (for a review, see Murray et al., 2015). The comparability of studies on neurocognitive development of IUGR children, is, however, limited due to the selection criteria for growth restriction. Unlike reduced growth in SGA fetuses, which is usually constitutional, the growth delay in IUGR has a pathological cause. We therefore identified IUGR fetuses by using ultrasound to estimate fetal weight as well as to measure the umbilical artery pulsatility index. The latter is a marker of placental blood supply and a clinical standard to monitor intrauterine malnutrition (Murray et al., 2015). In a follow-up sample of 83 very-low-birth-weight infants, Leppanen et al. used the mental scale of BSID-II to show that only the subgroup with a pathological Doppler was affected by an altered cognitive outcome at the age of 2 years, whereas motor development remained unaffected (Leppanen et al., 2010). This is akin to the present study: PDI of BSID-II did not reveal any differences in psychomotor development between IUGR, SGA, and AGA children at 2 years of age. Several other studies investigating motor outcomes in IUGR children also reported that no differences were observed (Wienerroither et al., 2001; Eixarch et al., 2008; Padilla et al., 2010). Some study results indicate an influence of prematurity and severity of IUGR on motor development (Gazzolo et al., 1995; Padilla et al., 2011).

The MDI of the BSID-II includes measures for different cognitive skills, i.e., active and passive speech development, problem solving, or memory performance. The updated version “Bayley Scales of Infant and Toddler Development, Third Edition” (BSID-III) provides more specific subscores: a cognitive scale, a receptive language and an expressive language scale. To establish specific approaches to support affected infants and their families with early interventions, the assessment should be performed with the updated version in future investigations. However, the German version of the third edition was not available at the time of this study, nor is a behavioral scale, as provided by the original versions of BSID-II and BSID-III, available in the German language to date. Results of studies investigating behavioral changes in former IUGR children indicate that attention, social-interactive skills or mood might also be affected (Roza et al., 2008; Beukers et al., 2017).

The major limitation of the current study is the low sample size, particularly for the IUGR group. Future studies with larger sample sizes should consider co-factors such as onset, duration and severity of IUGR to gain more detailed information about the impact of different types of IUGR (Miller et al., 2016). Moreover, loss to follow-up might be influenced by socioeconomic or demographic factors and might therefore bias our results (see **Figure 1** for drop-out at the different stages).

## Conclusion

The results of this study support the hypothesis that IUGR might be a risk factor for a delay in neurocognitive development (MDI) in two-year old children. However, the differences were only modest, and not significant with respect to the PDI, and the three study groups did not differ significantly in fetal event-related brain activity. The investigation of underlying physiological processes and their impact on human brain development should be the focus of further research. Moreover, larger trials with a standardized definition of IUGR and well-defined outcome measures are required to identify factors that impact the role of IUGR on child development. These findings would be instrumental in developing specific treatment and support for the affected infants and their families.

## Author Contributions

JH, HP and IK-S conceived and designed the study. MW, JP-F, SB, and IK-S recruited the participants. JP-F, SB, and IK-S performed fetal ultrasound and Doppler measurements. JH, JK, and MW carried out fMEG measurements. CW conducted the physical examination of neonates. JK and JH performed Bayley testing. JH, AC, FS and HP analyzed the data and were responsible for statistics. JH, FS, HP and IK-S prepared the draft manuscript. All authors made substantial corrections for the final manuscript.

## Conflict of Interest Statement

The authors declare that the research was conducted in the absence of any commercial or financial relationships that could be construed as a potential conflict of interest. The reviewer AM and handling Editor declared their shared affiliation at the time of the review.

## Abbreviations

AER: auditory event-related responses
AGA: appropriate for gestational age
BSID-II: Bayley Scales of Infant Development, Second Edition
BSID-III: Bayley Scales of Infant and Toddler Development, Third Edition
fMEG: fetal magnetoencephalography
fT: femto Tesla
GA: gestational age
Hz: Hertz
ISI: inter-stimulus interval
IUGR: intrauterine growth restriction
MDI: Mental Development Index
ms: millisecond
PDI: Psychomotor Development Index
SGA: small for gestational age
VER: visual event-related responses
